# Reduced biological effect of e-cigarette aerosol compared to cigarette smoke evaluated *in vitro* using normalized nicotine dose and RNA-seq-based toxicogenomics

**DOI:** 10.1038/s41598-017-00852-y

**Published:** 2017-04-18

**Authors:** Linsey E. Haswell, Andrew Baxter, Anisha Banerjee, Ivan Verrastro, Jessica Mushonganono, Jason Adamson, David Thorne, Marianna Gaça, Emmanuel Minet

**Affiliations:** British American Tobacco R&D Centre, Regents Park Road, Southampton, SO15 8TL UK

## Abstract

Electronic cigarettes (e-cigarettes) use has increased globally and could potentially offer a lower risk alternative to cigarette smoking. Here, we assessed the transcriptional response of a primary 3D airway model acutely exposed to e-cigarette aerosol and cigarette (3R4F) smoke. Aerosols were generated with standard intense smoking regimens with careful consideration for dose by normalizing the exposures to nicotine. Two e-cigarette aerosol dilutions were tested for equivalent and higher nicotine delivery compared to 3R4F. RNA was extracted at 24 hrs and 48 hrs post exposure for RNA-seq. 873 and 205 RNAs were differentially expressed for 3R4F smoke at 24 hrs and 48 hrs using a pFDR < 0.01 and a [fold change] > 2 threshold. 113 RNAs were differentially expressed at the highest dose of e-cigarette aerosol using a looser threshold of pFDR < 0.05, 3 RNAs exceeded a fold change of 2. Geneset enrichment analysis revealed a clear response from lung cancer, inflammation, and fibrosis associated genes after 3R4F smoke exposure. Metabolic/biosynthetic processes, extracellular membrane, apoptosis, and hypoxia were identified for e-cigarette exposures, albeit with a lower confidence score. Based on equivalent or higher nicotine delivery, an acute exposure to e-cigarette aerosol had a reduced impact on gene expression compared to 3R4F smoke exposure *in vitro*.

## Introduction

In the past 10 years, electronic cigarettes (e-cigarettes) have emerged as a substitute for cigarette smoking with the potential to be less harmful than conventional cigarettes^1, 2^. Following a review of the evidence available in the literature, Public Health England reported that e-cigarettes were likely to be 95% less harmful than conventional cigarettes and concluded that the health hazard caused by vaping “is likely to be extremely low and certainly much lower than smoking”^3^. This view was further upheld by the Royal College of Physicians which indicated that although harm from long-term use of e-cigarettes cannot be excluded, it is likely to be substantially less than conventional cigarettes^4^. Nevertheless, since e-cigarettes were recently introduced on the market, epidemiological data regarding the risks associated with these products is lacking.

*In vitro* testing evidence, however, support the view that vaping causes less damage to cell systems when compared to smoking. Examples of robust *in vitro* assessment studies of novel nicotine delivery devices (heated tobacco products and e-cigarettes) have used a relevant cell model, a well-considered exposure strategy combined with holistic untargeted omics screens to comprehensively assess the biological perturbations^5, 6^. Such *in vitro-*based systems toxicology approaches have been driven by the increasing demand to replace the use of low-throughput animal testing with high- throughput *in vitro* and computational technologies. This evolution was outlined in the National Research Council's recent publication, “Toxicity Testing in the 21^st^ Century (Tox 21): A Vision and a Strategy”^7^. Recognizing the limitation of traditional regulatory toxicity testing, Tox21 was initiated as a US multiagency effort to improve the risk assessment of chemicals by building on human cellular *in vitro* models and high-content, high-throughput screening^8^. Notably, the latest phase of the Tox21 program has been exploring the suitability of organotypic cell culture and transcriptomics for the assessment of adverse health effects caused by chemicals. The benefit of this paradigm shift in toxicology was illustrated in a recent study conducted by Moffat and colleagues where toxicogenomics and traditional toxicology approaches were compared to assess the toxicity of a known smoke carcinogen, B[a]P. The authors highlighted that both methods were successful at predicting B[a]P-induced DNA-damage but that the added value of toxicogenomics was to give powerful insights into the genotoxic and non-genotoxic mode of action of the toxicant^9^.

Following the Tox21 approache, a series of studies have used microarray-based transcriptomics analyses to demonstrate reduced impact of heated tobacco aerosol on 3D human *in vitro* airway cells compared to burned tobacco^5, 10^. More recently toxicogenomics RNA-seq-based analyses were applied to the testing of e-cigarette aerosol and also demonstrated reduced impact on the transcriptome^6^. RNA-seq presents multiple advantages as it is not limited to the targets present on a microarray and gives absolute RNA abundance values with greater dynamic range than microarrays^11^.

Although current systems toxicology studies reported a lesser impact of e-cigarette and heated tobacco aerosols *in vitro* ^5, 6^, other toxicological studies using targeted endpoints reported potential for DNA damage, oxidative stress and cytotoxicity^12, 13^. These discrepancies arise from the use of different cell models, products/devices, exposure matrices, times, and doses. The selection of a dose for comparative studies of e-cigarettes verses conventional cigarettes is particularly critical due to the fundamental differences in the nature and chemistry of the aerosol from these products. The study of aerosol dose *in vitro* or *in vivo* is a highly specialized discipline often referred to as “dosimetry”^14^. Many dosimetry tools and technologies have been developed to characterize the emissions from tobacco and nicotine products tested *in vitro*. These include assessing the chemical composition and mass delivery of aerosols products at source, through the generation and delivering system and at exposure^15, 16^. They can be used to support quantification of aerosol emissions *in situ* but also to confirm delivery to cellular cultures. Machine smoking regimen is a key dosimetry parameter for aerosol generation for *in vitro* exposure at the air liquid interface (ALI). Recent *in vitro* studies have focused on the ISO smoking regimen, however it has been demonstrated that it under-represents the dose of toxicants compared to human smoking^17^. More intense smoking regimens were developed such as the Massachusetts and Health Canada Intense (HCI) which could better represent human toxicant exposure in product testing. Whilst the smoking regimen is relevant in terms of aerosol chemistry, elements such as the puff number and exposure time are also important dose parameters in comparative product assessment^14^. Thus, dose matching between combustible cigarette and e-cigarettes can be conducted based on deposited mass or puff numbers. This, however, does not account for the reported change in smoking behavior to titrate for serum nicotine when subjects switch products^18, 19^. Therefore, a sensible approach would be to conduct comparative product assessment using an intense smoking regimen that delivers a similar nicotine dose range across the tested products.

Dosimetry was considered in the design of the study described here. Our aim was to compare the transcriptional response of a reconstituted 3D human primary airway cell system (MucilAir™) exposed to aerosol from a commercially available e-cigarette (Vype ePen) and a scientific reference cigarette (3R4F). Aerosols were generated at the HCI^20^ smoking regimen for 3R4F and the recommended CORESTA method CRM81 which is a variant of the HCI for e-cigarettes^21^. Continuous exposure of MucilAir^TM^ was conducted at the air liquid interface for 60 minutes at matched nicotine doses for each test product. E-cigarettes aerosols were also delivered at a nicotine dose twice higher than 3R4F. Using RNA-seq transcriptomics profiling, coupled with enrichment analysis and downstream causal reasoning we demonstrated that a minimal differential gene expression is observed at the tested exposure conditions for equivalent or higher nicotine delivery from the e-cigarette compared to cigarette smoke.

## Results

### Dosimetry

**Figure 1:**
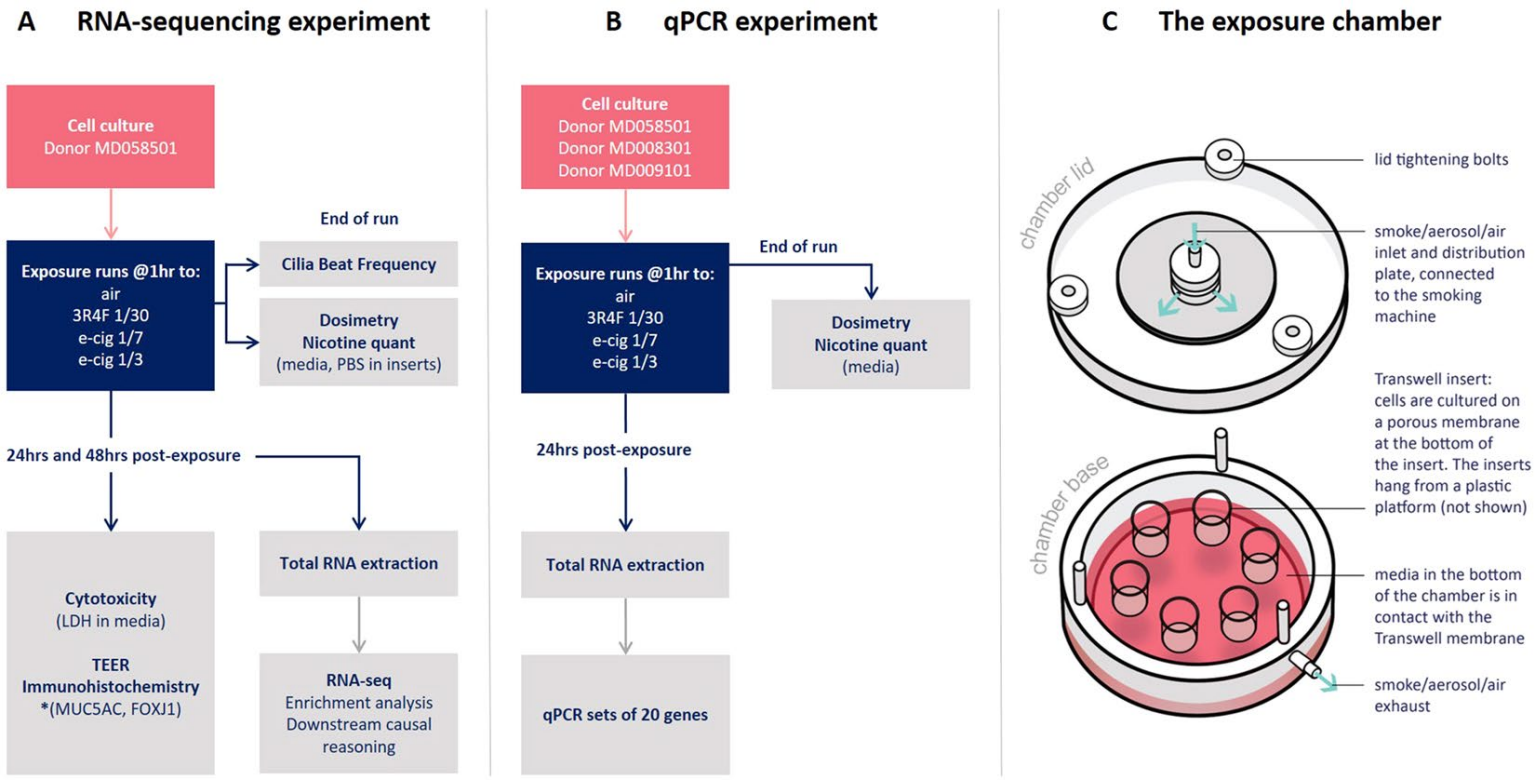
Schematic representation of the experimental design. The cells from one donor (#MD058501) were used for the RNA-seq experiment (**A**) and exposed to air, 3R4F smoke 1/30, e-cigarette aerosol 1/7 and 1/3. At the end of the exposure runs, nicotine was quantified from the media (1 media sample per chamber and run) and from 3 inserts filled with PBS for each treatment (**A**). CBF was also measured in the cell inserts immediately after exposure (**A**). The other endpoints (TEER, LDH) and RNA for RNA-seq were collected at 24 and 48 hrs post-exposure (**A**). For the RNA-seq experiment, three exposure runs were performed independently (Run1, 2, and 3) with 3 inserts for each treatment and time point. *Indicates that the immunochemistry were performed in a separate exposure experiment with donor #MD058501. Three independent runs with three inserts per treatment were also used. To validate the RNA-seq findings, a qPCR validation experiment was conducted with three donors (#MD058501, #MD008301, #MD009101) using the same exposure conditions and sets of 20 genes (**B**). The RNA was collected 24 hrs post-exposure (**B**). One exposure run was performed with 3 inserts per treatment. (**C**) Schematic of an exposure chambers with insert positions.

**Figure 2:**
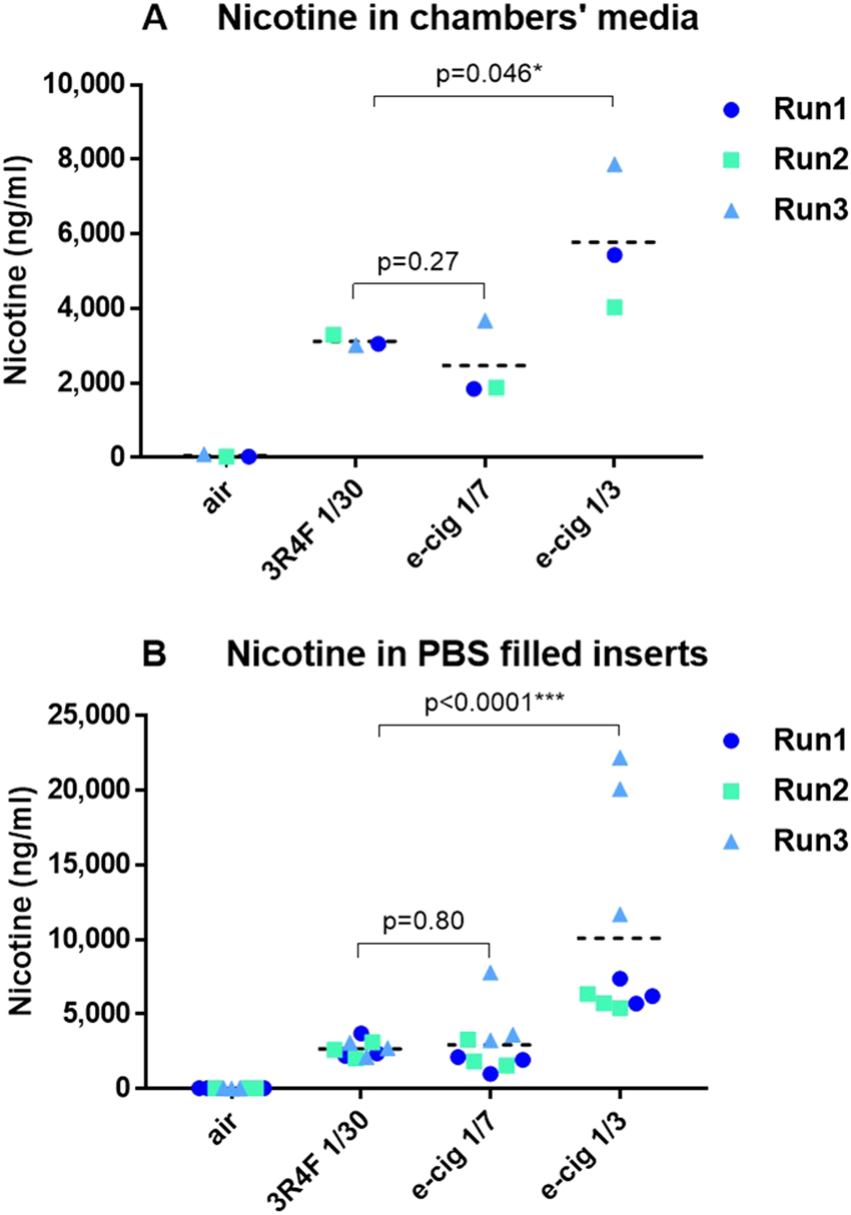
Scatter dot plot of nicotine delivered during the exposure runs in the basal media of the chambers (one measurement per chamber and run) (**A**) and in PBS filled inserts on the apical side (three inserts per chamber and run) (**B**). The nicotine value points for each independent experimental run (Run1, 2, 3) have been labelled in different colors. Three PBS filled inserts were used as technical replicates for apical nicotine quantification (**B**). The mean value is shown in each chart by the horizontal line. * and *** denote a t-test significance at p < 0.05 and p < 0.001, respectively, using 3R4F nicotine as comparator.

MucilAir™ differentiated at the ALI were exposed to air, 3R4F smoke diluted at 1/30, and the e-cigarette aerosol diluted at 1/7 and 1/3 with matching puff numbers (Fig. 1A). After exposure, the medium in the chamber and the PBS in dedicated inserts was collected for nicotine quantification (Fig. 1A,C). Figure 2A and B shows the concentration of nicotine recovered from each matrix per exposure run and per treatment. Nicotine delivery for 3R4F smoke at 1/30 (inserts 2742.5 ± 548 ng/ml, media 3124 ± 150 ng/ml) and the e-cigarette aerosol at 1/7 (inserts 2342.5 ± 947 ng/ml, media 2468 ± 1052 ng/ml) were the most consistent between runs whilst increased variability was observed for the e-cigarette aerosol at 1/3 dilution (inserts 8578.7 ± 5080 ng/ml, media 5778.9 ± 1940 ng/ml) (Fig. 2). This is likely to reflect the dilution operating limit of the smoke machine with the dense matrix delivered by e-cigarette. No statistical difference for nicotine delivery was observed between 3R4F smoke 1/30 and the e-cigarette aerosol 1/7 (inserts p = 0.27, media p = 0.8) (Fig. 2A and B). A statistically significant increase in nicotine delivery was observed when 3R4F smoke 1/30 was compared to the e-cigarette aerosol 1/3 (inserts p = 0.046, media p < 0.0001) (Fig. 2A and B). Thus, the analysis of nicotine from both the media and the inserts concurred to a nicotine delivery similar at 1/7 and higher at 1/3 for the e-cigarette compared to 3R4F at 1/30, respectively.

### Airway epithelia functional markers

Integrity of the tissue after treatments (air, 3R4F smoke 1/30, e-cigarette aerosol 1/7 and 1/3) was assessed using a series of functional respiratory epithelia and protein markers. These included (i) cilia beat frequency (CBF), (ii) trans-epithelial electric resistance (TEER), (iii) FOXJ1 and MUC5AC quantitative immunohistochemistry, and (iv) LDH release (cytotoxicity).

**Figure 3:**
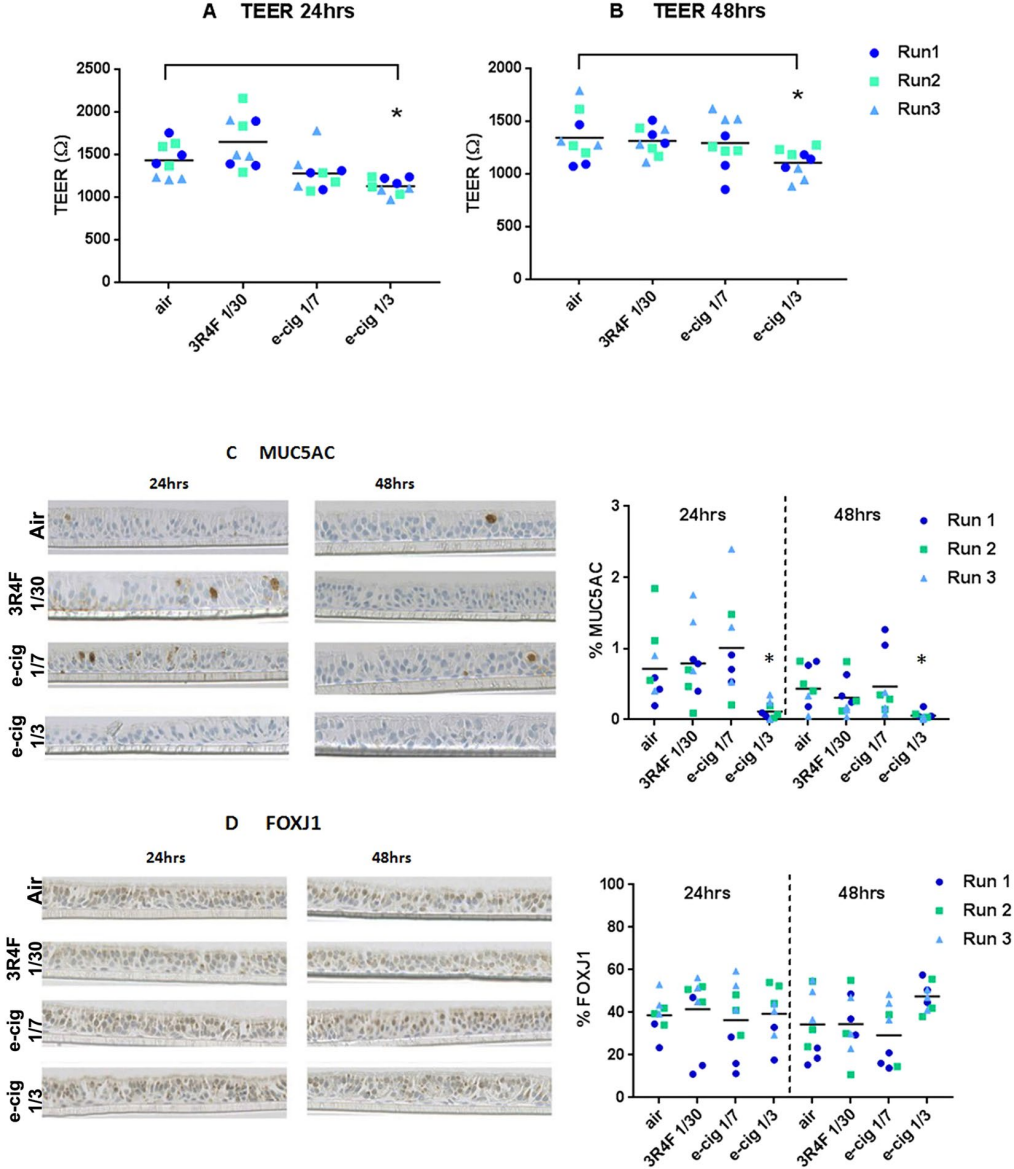
MucilAir™ functional markers after exposure to air, 3R4F smoke 1/30 and e-cigarette aerosol 1/7, 1/3. TEER at 24 hrs (**A**) and 48 hrs (**B**) post treatment. MUC5AC (**C**) and FOXJ1 (**D**) quantitative immunohistochemistry at 24 hrs and 48 hrs post treatment with the micrographies on the left and values plots on the right with the mean indicated by the horizontal line. *Denotes a significant difference versus air control at p < 0.05.
(iii) Quantitative immunohistochemistry for the marker of goblet cells MUC5AC and the marker of ciliated cells FOXJ1 are shown in Fig. 3C and D. The micrographs confirmed the differentiated pseudostratified tissue morphology with the presence of mainly ciliated cells and some goblet cells. MUC5AC quantitation (Fig. 3C) indicates a decrease (at p < 0.05) after exposure to 1/3 aerosol from the e-cigarette (24 hrs 0.1 ± 0.1%; 48 hrs 0.05 ± 0.5%) compared to air (24 hrs 0.7 ± 0.5%; 48 hrs 0.4 ± 0.3%). This result has to be taken with caution due to the limited representation of mucus producing cells, however, the % of mucus producing cell reported here is in agreement with what is observed in differentiated 3D airway cultures^23^. No significant change in FOXJ1 is observed at p < 0.05 (Fig. 3D).
(iv) Finally, cytotoxicity measured by LDH release, remained below 5% for all the time points and treatments (not shown).

(i) CBF was measured just after the exposure runs in all the inserts to detect any early acute cell damage (Supplementary Fig. S1). No reduction in CBF was observed between the air controls and the aerosol treated cells (Supplementary Fig. S1).
(ii) A significant TEER difference (p < 0.05) was found between air treatment (24 hrs 1432.7 ± 198 Ω; 48hrs 1342.9 ± 239 Ω) and the e-cigarette at 1/3 dilution (24 hrs 1130.9 ± 93 Ω; 48 hrs 1105.9 ± 130 Ω) (Fig. 3A and B), however, the values were still within a range indicating tight junctions’ integrity (600 Ω)^22^.

### Differential gene expression

**Figure 4:**
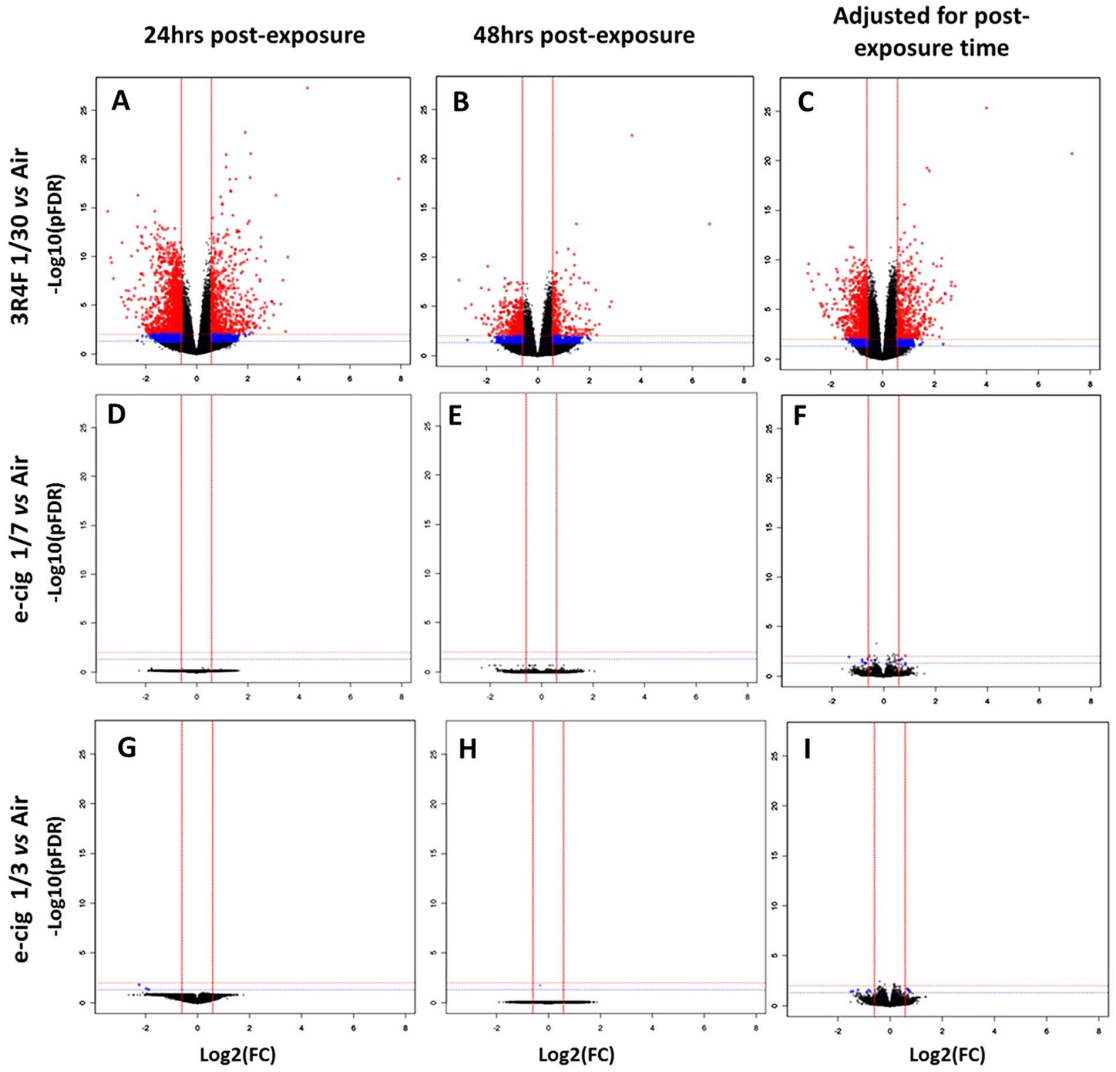
Volcano plots for the 9 RNA-seq contrasts. On top, air *vs* 3R4F 1/30 smoke dilution at 24 hrs (**A**), 48 hrs (**B**) post exposure, and air *vs* 3R4F 1/30 smoke adjusted for post exposure time (**C**). In the middle, air *vs* e-cigarette aerosol 1/7 dilution at 24 hrs (**D**), 48 hrs (**E**) post exposure, and air *vs* e-cigarette aerosol 1/7 dilution adjusted for post exposure time (**F**). At the bottom, air *vs* e-cigarette aerosol 1/3 dilution at 24 hrs (**G**), 48 hrs (**H**) post exposure, and air *vs* e-cigarette aerosol 1/3 dilution adjusted for post exposure time (**I**). The X-axis shows the Log2 of the fold change (FC) and the Y-axis shows the −Log10 of the adjusted p-values (pFDR). The dotted horizontal lines show the 0.05 pFDR threshold (blue line) and the 0.01 pFDR threshold (red line). The vertical red lines show the +1.5 and −1.5 fold change thresholds. Blue dots represent significant RNA features at pFDR < 0.05 and −1.5 > FC > +1.5 and red dots represent significant RNA features at pFDR < 0.01 and −1.5 > FC > +1.5.

RNA was extracted from MucilAir™ cells exposed to air, 3R4F smoke diluted at 1/30 and the e-cigarette aerosol diluted at 1/7 and 1/3 (Fig. 1A). All 72 samples passed the RNA extraction, RNA-seq library, and sequencing QC. Following alignment with the human reference genome (GRCh38) 48,854 RNA species were identified for the statistical analysis. The normalized RNA expression data was analysed in 6 contrasts relative to the air controls (3R4F *vs* air at 24, 48 hrs; e-cigarette aerosol 1/7 *vs* air at 24, 48 hrs; e-cigarette aerosol 1/3 *vs* air at 24, 48 hrs). To increase statistical power to pick up genes that might be responders, another 3 contrasts were performed adjusted for post-exposure recovery time (3R4F *vs* air, e-cigarette aerosol 1/7 *vs* air, e-cigarette aerosol 1/3 *vs* air), combining the 24 hrs and 48 hrs data. The differential expression results for all these contrasts are presented in 9 volcano plots (Fig. 4). Table 1 summarizes the number of up and down regulated RNA features for different thresholds. The strongest differential response was observed for 3R4F smoke at 24 hrs post-exposure (873 RNAs at pFDR < 0.01, [FC] > 2) followed by a recovery at 48 hours (205 RNAs pFDR < 0.01, [FC] > 2). No more than 3 RNAs were differentially expressed at 24 hrs and 48 hrs post exposure to e-cigarette aerosol compared to air (Table 1). When the dataset was adjusted for post-exposure time (combining 24 hrs and 48 hrs) and a pFDR < 0.05 was used, 113 differentially expressed RNAs were identified as responsive to the highest concentration of e-cigarette aerosol but only 3 exceeded a fold change of 2 (Table 1). Another 49 RNAs were identified to be responsive to the more diluted 1/7 dose of e-cigarette aerosol at pFDR < 0.05. The full list of RNAs is given in Supplementary Tables S1 to S9. 20 RNAs species overlapped between the two e-cigarette aerosol dilutions at pFDR < 0.05 with adjustment for time (Supplementary Fig. S2). 11 genes from the e-cigarette aerosol exposure data also overlapped with the 3R4F smoke treatment adjusted for post-exposure time at pFDR < 0.01, [FC] > 2 (Supplementary Fig. S2).

**Table 1:** Number of differentially expressed RNA features for each treatment for different pFDR and FC thresholds.

	pFDR<	FC>	24 hrs		48 hrs		24 and 48 hrs combined (adj time)	
			up	down	up	down	up	down
3R4F 1:30	0.05	1	3598	4199	2449	2184	4003	4194
	0.05	2	422	997	198	200	267	310
	0.01	1	2556	2690	1567	1204	2923	2951
	0.01	2	318	555	86	119	223	240
e-cig 1:7	0.05	1	0	0	0	0	28	21
	0.05	2	0	0	0	0	0	1
	0.01	1	0	0	0	0	6	3
	0.01	2	0	0	0	0	0	0
e-cig 1:3	0.05	1	0	3	0	1	49	64
	0.05	2	0	3	0	0	0	3
	0.01	1	0	0	0	0	3	2
	0.01	2	0	0	0	0	0	0

Supplementary Materials (attached separately).

### Gene enrichment analysis and downstream causal reasoning

**Figure 5:**
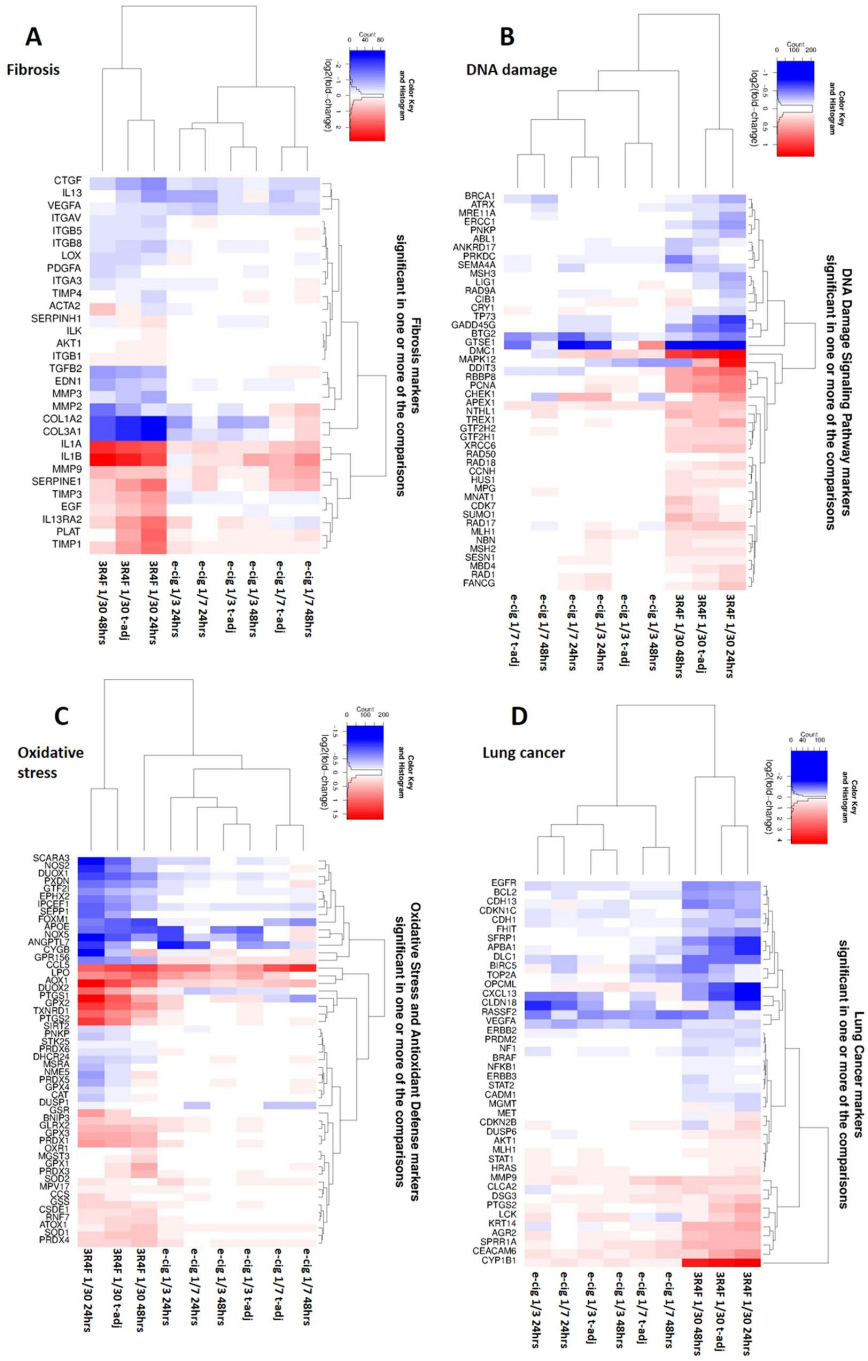
Unsupervised hierarchical clustering for Fibrosis (**A**), DNA-damage (**B**), Oxidative Stress (**C**), and Lung cancer (**D**) genesets. Gene markers significant at pFDR < 0.05 in one or more of the treatment contrasts are shown. Red indicates up-regulated genes and blue is used for the down-regulated genes. The color intensity is a function of the Log2 (fold change).

The RNA-seq profiling data was mapped to a collection of 131 pathway-focused genesets to identify enriched expression of genes involved in specific biological function and disease processes. Heatmaps were generated for the 9 contrasts (product treatment *vs* air at 24 and 48 hrs and adjusted for time). The heatmaps present fold-changes for RNAs significant at pFDR < 0.05 found in at least one or more of the contrasts. The contrasts clustered based on similarity of expression patterns. In Fig. 5 we present the results for four genesets relevant to the disease processes associated with smoking: Fibrosis (**A**), DNA damage signalling (**B**), oxidative stress response (**C**), and lung cancer (**D**). Clear clusters are observed for the 3R4F smoke treatment and a second cluster is formed by the e-cigarette aerosol treatments. The largest fold change variations were clearly observed across all 4 genesets for the 3R4F smoke treatment *vs* air (Fig. 5). The data for the 131 genesets is provided in Supplementary Table S10.

**Figure 6:**
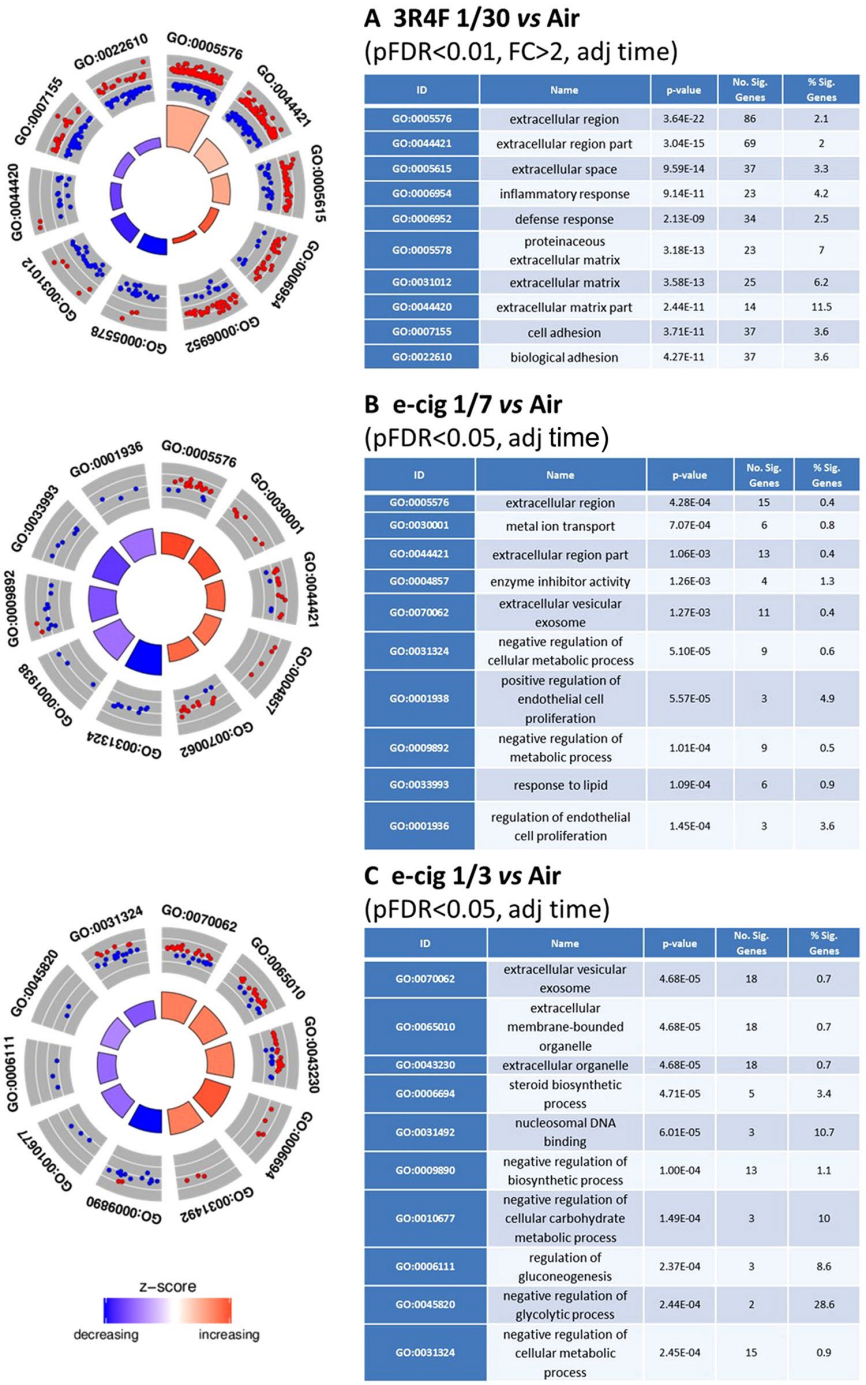
GO enrichment circle plots for the top five most significant GO categories with positive and negative z-score for each treatment adjusted for time. 3R4F 1/30 smoke exposure (**A**), e-cigarette aerosol exposure at 1/7 (**B**) and 1/3 (**C**) dilutions are presented with the statistical filter used to select the genes included in the enrichment. The outer circle shows the relative fold change for each significant RNA feature compared to air contributing to the GO term with blue dots showing down-regulated RNA features and red dots showing up-regulated features. The inner quadrants are colored based on the z-score and their surface is a function of the enrichment p-value, the larger the surface the lower the p-value. The z-score color scale represents the number of up- regulated genes minus the number of down- regulated genes for a given GO term divided by the square root of the total count. The associated tables present the GO term ID and function, p-value for the enriched term, and the number of RNA features contributing to the GO term enrichment.

Significant genes at pFDR < 0.01 and [FC] > 2 for 3R4F and pFDR < 0.05 for e-cigarette aerosol exposure from each contrast adjusted for post exposure recovery time were also analysed for enrichment of GO (Gene Ontology) terms across all three GO categories using a hypergeometric test. The complete list of significant GO terms (at p < 0.01) enriched for up- or down-regulated genes *vs* the air control is given for each treatment in Supplementary Tables S11 to S18. The GO plots for top enriched GO terms based on p-values are shown in Fig. 6. These plots show a circular representation of the relative fold-changes of genes compared to the air control within the top up/down enriched GO terms. For 3R4F the top enriched GO terms indicate perturbations in the extracellular matrix components, inflammatory and stress response with significance p-values ranging from 3.7e^−22^ to 2.1e^−9^ (Fig. 6A). For the e-cigarette (1/3 and 1/7), the top enriched categories based on p-values ranging from 4.68e^−5^ to 1.27e^−3^ indicate differential expression of genes involved in metabolic/biosynthetic processes, the transformation of chemical substances, glycolytic processes and changes in the extracellular membrane (Fig. 6B,C). The GO terms ID found in common in our two e-cigarette aerosol dilutions compared to air relates to extracellular membrane processes and negative regulation of metabolic/biosynthetic processes and are listed in Supplementary Table S11. Other GO terms relating to apoptosis and hypoxia found in our enrichment (Supplementary Tables S14 to S18) were of interest since they were reported in a previous work^6^ with e-cigarette and this will be examined further in our discussion.

The direction of the fold change (up, down) for the genes used in the GO enrichment analysis was used for causal reasoning with IPA ingenuity® to predict downstream effects on biological functions. Thirty-one disease and functions were predicted to be increased with cigarette smoke exposure with a z-score greater than 2 (Supplementary Table S19). Four of those associated functions had a p-value below 1.e^−08^, namely inflammatory response, growth of tumor, activation of granulocytes, and angiogenesis (Supplementary Table S19). The low enrichment p-values and [z-score] > 2 indicate a high degree of confidence in this prediction obtained from a robust set of genes selected at pFDR < 0.01 and [FC] > 2. Overall, a few categories with z-scores below -2 or above 2 were recorded for the e-cigarette aerosol exposure from a set of genes that was selected based on a pFDR < 0.05. Seven functions had a [z-score] > 2 of which 6 (eg: cellular homeostasis, apoptosis of different cell types) were predicted to be down-regulated for e-cigarette aerosol exposure at 1/7 dilution (Supplementary Table S19). Three functions were predicted to be up-regulated (z-score > 2) for the 1/3 e-cigarette aerosol dilution relating to cell movement and the metabolism of lipids and terpenoids (Supplementary Table S19). The enrichment p-values for those predicted functions were overall higher than for cigarette smoke indicating a lower confidence (Supplementary Table S19).

### qPCR validation in multiple donors

**Figure 7:**
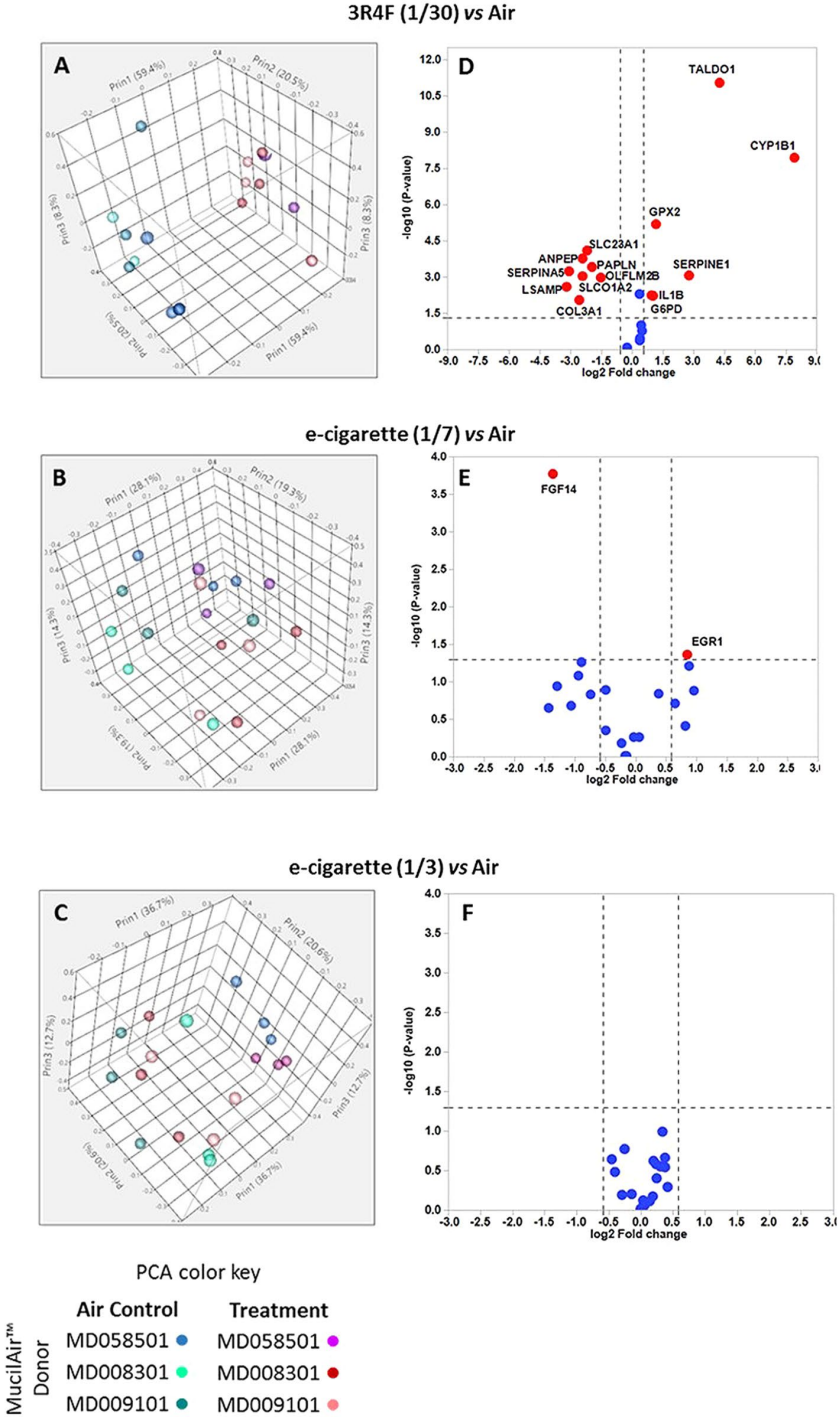
qPCR validation of RNA-seq data in MucilAir™ cells from 3 different subjects at 24 hrs post-exposure. Sets of 20 genes per treatment were selected from the RNA-seq data for qPCR screening in MucilAir™. PCA plots of qPCR data comparing treatments (air vs 3R4F 1/30 (**A**), e-cigarette 1/7 (**B**) and e-cigarette 1/3 (**C**)) and subjects (#MD058501, #MD008301, #MD009101). Corresponding volcano plots showing the response for each individual gene using the data from the three donors (3R4F 1/30 (**D**), e-cigarette 1/7 (**E**) and 1/3 (**F**)). Dots colored in red indicate differentially expressed genes significant at p < 0.05 and with a FC > [1.5]. The red dots are labelled with the gene name.

Since the RNA-seq data was obtained from tissue cultures from one donor (#MD058501), we further validated our results by qPCR in MucilAir™ from multiple donors (#MD058501, #MD008301 and #MD009101). A new independent exposure run was performed and RNA was extracted at 24 hrs post-exposure for qPCR. Sets of 20 genes significant at least at pFDR < 0.05 were selected for qPCR from the 3R4F 1/30, e-cigarette 1/7 and 1/3 RNA-seq data (Supplementary Table S20). Principal component analysis performed on the qPCR data clearly separated the response from 3 donors exposed to 3R4F 1/30 smoke from the air controls indicating a distinct qPCR profile for this geneset and treatment (Fig. 7A). No separation was observed based on the qPCR results comparing the air controls and the e-cigarette exposure at 1/3 and 1/7 dilutions (Fig. 7B,C). A detailed analysis showing the gene expression fold changes and significances for each donor (Supplementary Table S21) and the combined data for all 3 donors (Supplementary Table S21 and Fig. 7D,E,F) confirmed that there is a good agreement between the 3R4F RNA-seq and the qPCR data. For the three donors combined, 14 genes out of 20 were differentially expressed at p < 0.05 and FC > [1.5] all of which were matched for up or downregulation with the 3R4F 1/30 RNA-seq data (Supplementary Table S21, Fig. 7D). The absence of response from CYP1A1 was unusual and was not investigated further as no RNA/cDNA template was left from these donors. However, as part of an experiment that we conducted for another study with a different MucilAir™ donor (#MD059401) the exposure to 3R4F smoke was repeated using the same run conditions and RNA-seq was performed. We observed CYP1A1 induction between 1348- and 876-fold at pFDR < 0.01 at 24 hrs and 48 hrs, respectively. We also previously reported CYP1A1 induction by cigarette smoke in MucilAir™ in shorter exposure runs^24^. Finally, for the e-cigarette aerosol treatment, only FGF14 and EGR1 were significant in the qPCR at p < 0.05, FC > [1.5] at the 1/7 aerosol dilution (Supplementary Table S21, Fig. 7E,F).

## Discussion

The toxicological assessment of e-cigarettes has so far reported fewer biological responses on cellular functions compared to conventional cigarettes^6, 12, 25–27^, however, approaches for dose and matrices have been inconsistent. Yu and colleagues compared e-cigarette aerosol extracts with cigarette smoke extracts generated in media applied to both primary and immortalized epithelial cells. The authors concluded that “vaporized e-cig liquids induce increased DNA strand breaks and cell death”^13^. The study did not report, however, whether the media preparation was quality checked for dose and different exposure times were used for e-cigarette- and cigarette-treated media. Whole aerosol exposure of cells grown at the ALI is potentially a better model as it replicates the normal exposure route and chemistry of the aerosol matrix. This type of approach was employed by Scheffer and colleagues to expose primary airway cells to e-cigarette aerosol and cigarette smoke using a reference ISO smoking regimen^12^. No dosimetry assessment other than puff matching was included in the study to compare dose of aerosol reaching the cells. As the authors indicated the number of puffs taken by users of e-cigarette and conventional cigarettes can vary considerably^12^, therefore puff matching might not be the most relevant dosimetry anchor for *in vitro* testing. In our recent e-cigarette studies, we have shown the importance of expressing dose as a function of delivered nicotine, when conducting comparative studies across different tobacco and nicotine product categories. Azzopardi *et al*.^25^ and Adamson *et al*.^7, 28^ showed how deposited mass and nicotine quantification at the cellular exposure interface enable appropriate extrapolation and comparisons in an aerosol matrix. Different types of e-cigarettes and e-liquids generate different volumes of aerosol and deliver different amounts of nicotine, however typically smokers who switch from cigarette to nicotine delivery devices adapt their behavior to potentially titrate for nicotine^18, 19^. With this being considered, Shen *et al*. reported that primary airway cells exposed to whole e-cigarette aerosol generated at ISO smoking regimen had a lesser impact on differential gene expression than combustible cigarette^6^. The authors indicated that “approximately equivalent dosing of cells” was used, the dosimetry data, however, was not presented^6^.

Here, we used the learnings from these different studies to (i) generate the aerosol using a more intense smoking regimen (HCI), and (ii) used two e-cigarette aerosol dilutions based on the nicotine delivered to an organotypic airway cell systems. For our exposure runs, we selected the HCI smoking regimen^20^ for 3R4F and CORESTA CRM81 variant of the HCI regimen for e-cigarettes^21^ which is of higher intensity than the ISO smoking regimen^17^. Dose comparison between cell treatments was based on nicotine measured in the exposure chamber inserts and media during the smoking runs (Fig. 2). One e-cigarette aerosol dilution (1/7) was used to deliver equivalent nicotine compared to 3R4F 1/30. An additional 1/3 e-cigarette dilution was tested where the average level of nicotine delivered to the cells was approximately doubled compared to 3R4F cigarettes 1/30 (Fig. 2). The 1/30 dilution for cigarette smoke was selected because it has been estimated that this is within the range of dilution observed through normal inhalation in smokers^29^. The same number of puffs was used between products. No statistical difference was observed between the average nicotine level delivered from 3R4F smoke at 1/30 dilution and e-cigarette at 1/7 dilutions, whilst the level of nicotine is approximately double for a 1/3 e-cigarette aerosol dilution (Fig. 2). Greater nicotine delivery variability, however, was observed from run to run for e-cigarette compared to cigarette smoke, especially in cell free inserts placed in the exposure chambers for run number 3 dilution 1/3. This is possibly due to the heavier, stickier nature of the e-cigarette aerosol and variability in dilution performance at the higher aerosol concentration in this run. Since there was relatively good consistency in the level of nicotine across all runs measured directly in the exposure chambers media no samples were excluded. Furthermore, Margham *et al*. reported a nicotine dose per puff of 0.032 mg of nicotine for the Vype ePen and 0.17 mg for 3R4F smoked at a variant of the HCI regimen (CORESTA CRM81), and HCI, respectively. Based on these numbers there is overall a good agreement between the data from Margham and colleagues^30^ and the dilution ratio we used for 3R4F and Vype ePen to achieve nicotine dose matching and one e-cigarette dose exceeding 3R4F nicotine by approximately two-fold.

After one-hour exposure, different endpoints were investigated at 24 hrs and 48 hrs post treatment, including total RNA for differential RNA-seq profiling. Morphological and functional markers assessed by immunohistochemistry, TEER, CBF, and LDH-release only reported limited impact from treatments (Fig. 3 and Supplementary Fig. S1). Initial testing indicated that higher 3R4F smoke concentrations (1/20) have a marked deleterious effect on some of those markers such as CBF and the % of ciliated cells and cytotoxicity (not shown), hence we did not use a higher smoke concentration.

Differential gene expression analysis by RNA-seq revealed a striking difference between products treatment at 24 hrs and 48 hrs post-exposure with several hundred responsive RNAs at pFDR < 0.01 and [FC] > 2 for cigarette smoke *vs* air treatment (873 RNAs at 24 hrs and 205 at 48 hrs) (Table 1, Fig. 4). Up to 113 and 49 differentially expressed RNAs could be identified for the e-cigarette aerosol dilutions 1/3 and 1/7 but only after increasing the pFDR threshold to 0.05, excluding the fold change filter and adjusting for post-exposure time, effectively merging the 24 hrs and 48 hrs dataset to increase statistical power (Table 1, Fig. 4). When the e-cigarette data was not adjusted for post-exposure time, only 1 and 3 genes (pFDR < 0.05) were significantly decreased at the highest aerosol concentration (1/3) at 24 hrs and 48 hrs, respectively (Table 1). These results were in agreement with other transcriptomics studies using e-cigarette aerosols. Notably, in a study conducted at the air liquid interface with human bronchial epithelial cells and using an ISO smoking regimen, 9 genes were reported to be differentially expressed at 24 hrs post exposure to e-cigarette aerosol with nicotine (qFDR < 0.05) down from 47 genes at 1 hour post exposure (qFDR < 0.05)^6^. In another acute exposure study performed with BEAS-2B cells exposed at the air liquid interface no genes were differentially expressed 24 hrs after exposure to unflavored e-cigarette aerosol with nicotine compared to air, and 5 genes were upregulated when a tobacco flavored e-liquid was used^31^. It is important to note that in this latest study the pFDR and fold change thresholds used were more restrictive (pFDR < 0.01 and FC > 2)^31^.

Genesets enrichment (threshold pFDR < 0.05) and gene ontologies (GO) enrichment (thresholds pFDR < 0.01, [FC] > 2) analyses identified changes in the expression of genes involved in tissue remodelling, cell adhesion (chemotaxis), oxidative stress, inflammation and DNA damage and repair after exposure to 3R4F cigarette smoke (Figs 5, 6, Supplementary Tables S10 and S12–S13) amongst others. Changes in those processes are in good agreement with numerous studies on cigarette smoke toxicity conducted *in vitro* and *in vivo* and models of tobacco-related lung diseases including cancer and COPD^32–34^. The low p-values obtained for the GO enrichment indicate that the over-represented categories are not due to chance and are based on a substantial number of gene hits (Fig. 6, Supplementary Tables S12–S13).

In contrast, treatment of MucilAir™ with the e-cigarette aerosol matched or in excess of the nicotine delivered by a combustible cigarette did not yield a strong differential RNA expression compared to air at the two time points tested (Table 1, Fig. 4). By adjusting for the post-exposure time, and loosening the thresholds with no fold change filter and a pFDR < 0.05, 49 and 113 differentially expressed RNAs compared to the air control were detected at the 1/7 and 1/3 aerosol dilution, respectively (Table 1, Supplementary Tables S8–S9). These genes were used to conduct an enrichment analysis for e-cigarette aerosol compared to air. A moderate proportion of the top up-regulated and down-regulated GO terms overlapped between the 1/7 and 1/3 aerosol dilution and were mainly related to extracellular membrane components and negative regulation of metabolic/biosynthetic processes (Fig. 6, Supplementary Table S11).

Overall our results are in agreement with another transcriptomics study that also reported less biological responses from e-cigarette aerosols in cultures of human bronchial epithelial cells 24 hrs after exposure^6^. In that study, an acute exposure was performed at the air liquid interface using an e-cigarette vaped at the ISO smoking regimen which is less intense than the HCI smoking regimen we used. The authors indicated that at 24 hrs post exposure to e-cigarette aerosol with nicotine no functional annotation cluster analysis (GO) could be performed due to the low number of gene candidates^6^. However, when the same authors analysed the transcriptional profile 1 hr post-exposure to the same e-cigarette aerosol they were able to report functional enrichment for cell cycle, hypoxia, inflammation, response to organic substance, biosynthetic processes, and apoptosis based on 47, mostly downregulated genes^6^. Further perturbations of genes involved in phospholipids and fatty acid metabolism were also reported at multiple time points post exposure and the authors proposed that this could be due to glycerol exposure^6^. Our study did not include a short post-exposure recovery time and therefore no direct comparison was possible. Both studies included a 24 hrs post-exposure time point but this time point did not yield sufficient gene candidates to perform a gene ontology analysis. However, using the list of genes from our e-cigarette treatments (1/7 and 1/3) adjusted for time (pFDR < 0.05, no FC) and comparing it to the enriched GO terms from Shen and colleagues^6^ e-cigarette at 1 hr time point a total of 33 enriched gene ontology terms were found in common (Supplementary Table S18). Those included negative regulation of metabolic/biosynthetic processes, response to organic substances, apoptosis, and hypoxia. Although the fold changes from our e-cigarette genes list from combining the 24 hrs and 48 hrs time point were in general below 2 (Table 1 and Supplementary Tables S8–S9), it is possible that, as reported by Shen *et al*.^6^, an early acute response took place with fold changes of greater magnitude. Therefore, shorter time points should be considered in future studies.

Caution is advised in the interpretation of the biological impact of the expression changes in the highlighted pathways since enrichment does not predict whether a pathway is activated or inhibited. Hence downstream causal reasoning approaches can add an extra layer of granularity in the interpretation of enrichment using the direction of the fold change to predict the outcome on a given pathway. Our downstream causal analysis using IPA ingenuity®^35^ predicted an increase (z-score > 2) in inflammation, cell movement (chemotaxis), and tumor related pathways (Supplementary Table S19) for cigarette smoke exposure with differential RNAs selected at the pFDR < 0.01 and [FC] > 2 thresholds. A decrease (z-score <-2) in apoptosis and cellular homeostasis, and increase (z-score > 2) in elements of lipid metabolism, and cell movement was highlighted for e-cigarette exposure (Supplementary Table S19). It is important to note that such analyses are useful to propose testable hypothesis and should be considered in the context of designing more targeted studies to verify the validity of the prediction. Such assays could include, for example, confocal high-content screening for adipogenesis, apoptosis, oxidative stress, and mitochondrial activity applied to the epithelium exposed at the ALI.

A limitation of our study was the use of one subject to conduct most of this work at an acute exposure regimen and therefore we cannot expand our conclusion to different individuals and longer exposure conditions. In a recent report, the gene expression profile of 4 MucilAir™ donors exposed to 3R4F smoke was compared and a 0.76 Pearson Correlation coefficient was calculated, indicating a high degree of agreement in the gene response profile between donors^36^. Donor to donor variability could affect the statistical power of the gene expression analysis, especially in the e-cigarette treatment group where a weak response is observed. To power such experiment, an increased number of inserts and repeat could be used from additional donors, but only a finite number of inserts can be obtained for each donor, thus this can be limiting if multiple time points, end points, and aerosol dilutions are tested. With this in mind, we addressed the single donor limitation of our study by performing a qPCR validation of representative genes selected from the RNA-seq data using cells from three different subjects. The qPCR results clearly confirmed that 3R4F smoke triggers a robust transcriptional response when different donors are tested with 14 out of 20 RNAs in agreement with the RNA-seq experiment (Fig. 7). The qPCR geneset selected from the 3R4F RNA-seq data was quite robust since they satisfied the stringent threshold criteria of pFDR < 0.01 and [FC] > 2. It was only possible to select qPCR genes from the e-cigarette RNA-seq data by lowering the threshold to pFDR < 0.05 with no FC filter which can lead to the inclusion of false positives. This was reflected in the qPCR results with only a few genes showing agreement between e-cigarette NGS and e-cigarette qPCR (Supplementary Table S21).

It is worth noting that out of the few transcriptomic studies that have currently been performed with *in vitro* smoke exposure or with clinical samples from smokers, the degree of overlap between the responsive genes can be limited. Cross-comparisons between transcriptomic studies from Beane *et al*., Chari *et al*., Hackett *et al*., Mathis *et al*., Shen *et al*.^6, 37–40^ and the work presented here only show a 3–6% gene overlap. This lack of consensus can be attributed at least in part to the design of the studies with cells coming from either the upper or lower respiratory tract, different smoking doses and regimens. In some instances, the RNA was extracted directly from cells obtained by brushing or biopsies of the respiratory epithelium of smokers^35–37^, whilst other have used organotypic tissue cultures exposed to machine-generated smoke^6, 38^. Even if these overlaps can be considered moderate at best, the resulting enrichment analyses appear more consistent with apoptosis, inflammation, cell cycle, and xenobiotics metabolism typically being the top over-represented categories. This indicates that the differential expression of a collection of responsive genes is more informative than a gene taken in isolation.

Therefore, we can conclude that the data strongly supports the adverse effect of acute exposure to cigarette smoke on MucilAir™ cells with functional enrichment for cancer, inflammation and fibrosis genes. In contrast, RNA-seq-based toxicogenomics showed a reduced impact of e-cigarette aerosols acute exposure on MucilAir™ cells compared with 3R4F reference cigarette at equivalent or higher dose of nicotine exposure. However, exposure to e-cigarette aerosol is not neutral compared to air with changes in genes mapping to metabolic/biosynthetic processes, apoptosis, hypoxia and extracellular membrane pathways. The small amplitude of the alteration in genes expression following e-cigarette aerosol exposure warrants further pathway focused studies at early, intermediate and late time points to confirm the biological relevance of those highlighted changes. Additional studies should also include repeated exposure over multiple days to better evaluate the long-term effects of e-cigarette aerosols.

## Materials and Methods

### Cell culture

MucilAir™ (Epithelix Sarl, Geneva, Switzerland), a reconstituted human airway epithelia differentiated at the ALI was used for this project. Three donors (non-smokers) were used: donors #MD058501, age 41, Caucasian female; #MD008301 age 61, Caucasian male; #MD009101, age 48 Caucasian female. For the initial exposure experiment, including RNA-seq, donor #MD058501 was used; all three donors were used for subsequent qPCR experiments. The cells were isolated at Epithelix Sarl from nasal swabs after obtaining permission by informed consent in compliance with the declaration of Helsinki on biomedical research, and approval from local ethics commission to be used for research purpose. The cells are then passaged at Epithelix Sarl to create sufficient material before seeding and differentiation on the inserts and shipment. Passaged cells *in vitro* are not deemed relevant material under the UK Human Tissue Act (HTA) 2004 which regulates storage, use and disposal of human tissue^41^. The cells were used for research purpose only and the culture were supplied fully anonymized.

### Products

3R4F reference cigarettes (University of Kentucky) are a 9.5 mg ISO tar yield tobacco product. 3R4F were conditioned in accordance with ISO 3402:1999^42^ before use. The commercially available Vype ePen e-cigarette (Nicoventures, UK) is a closed modular device and the ‘Blended Tobacco’ variant was used containing18 mg/ml nicotine replaceable e-liquid cartridge composed of 25% (w/w) propylene glycol, 48.14% vegetable glycerine, 25% water, and (<1%) blended tobacco flavor^30^.

### Experimental design and exposure method

Smoke from 3R4F cigarettes was generated using a RM20S smoking machine (Borgwaldt KC, Germany) under the HCI smoking regimen (55 ml puff volume drawn over 2 s once every 30 s with a bell-wave puff profile (the profile shape reflects the smokers’ puffing behavior)^20, 43^. Aerosol from the e-cigarette was produced using the RM20S smoking machine under the CORESTA method n°81 (CRM81), a variant of the HCI regimen recommended for e-cigarettes (55 ml puff volume, 3 s puff duration, 30 s puff interval, and a square wave puff profile)^21^. MucilAir™ inserts were exposed in acrylic exposure chambers for 1 hr to cigarette smoke at 1/30 dilution (aerosol:air, v-v) or e-cigarette aerosol at 1/3 or 1/7 dilution (aerosol:air, v-v). The number of puffs was matched between products. Air controls were performed with cells exposed to an intermittent flow of sterile air in an exposure chamber, at a frequency, volume identical and duration as treated cells. For RNA-seq, the exposures were repeated 3 times independently (Run 1, 2 and 3) and each experiment included 3 intra-experimental replicates with donor #MD058501. For qPCR, cells from three subjects were used in one exposure run and with 3 intra-experimental replicates. After exposure cells were incubated in fresh 700 μl MucilAir™ Culture Medium for 24 (qPCR and RNA-seq) and 48 hrs (RNA-seq). The basal media was collected for LDH measurement. The cells were lysed and stored at −80 °C for total RNA extraction. A schematic of the experimental design is presented in Fig. 1.

### Nicotine quantification

The concentration of nicotine was determined at completion of the exposure from cell culture media collected in the exposure chambers and cell-free inserts with PBS placed in the chambers. The media and PBS were spiked with d4-nicotine (Sigma Aldrich, St Louis, MO, USA) at a final concentration of 10 ng/ml. The samples were evaporated under a vacuum and the dried residues were dissolved in 1 ml 5% acetonitrile:water (v/v). A Waters Acquity UPLC (Waters, Milford, MA, USA) connected to an Applied Biosciences 4000QTrap mass spectrometer (Applied Biosystems, Foster City, CA, USA) was used for the quantification of nicotine. The UPLC method was adapted from Onoue *et al*.^44^ and the quantitative MS/MS method used the settings described by Jin *et al*.^45^.

### Functional and immunohistological endpoints

(i) The CBF was measured 24 hrs and 48 hrs post-exposure using an inverted AX10 Zeiss microscope (100x magnification, 10x ocular, 10x objective) (Zeiss, Gottingen, Germany) fitted with a heated chamber (Pecon, Erbach, Germany) maintained at 37 °C and a high speed digital camera^46^ (Ammons engineering, Clio, MI, USA). Immediately after exposure, MucilAir™ cultures were equilibrated for 5 min prior to measurement and video was recorded at 120 frames per s, capturing at least 512 frames.

(ii) Data on TEER was collected 24 and 48 hrs after exposure by adding PBS to the apical surface and measured with a resistance meter (World Precision Instruments, Florida, USA).

(iii) The immunohistochemistry were conducted after fixing the cell inserts with 3.6% formaldehyde/PBS, followed by a dehydration step using 70% ethanol and paraffin inclusion. Cells were fixed 24 and 48 hrs post-exposure. Tissue sections (4 µm) were incubated with the MUC5AC and FOXJ1 primary antibody (Abcam, Cambridge, MA, USA) for 1 hr at room temperature. After 3 washes the biotinylated secondary antibody (Dako, Ely, UK) was used to detect the primary, and visualized using an HRP-conjugated ABC system. Quantitative image analysis was performed using ImagePro Plus6.2 (Media Cybernetics, Cambridge, UK). Twenty images spanning the entire length of the section were acquired per insert to obtain a composite % for MUC5AC and FOXJ1.

(iv) Cell viability was determined at the different time points via LDH release measured in the culture media using the CytoTox-One assay (Promega, La Jolla, CA, USA).

### RNA isolation

Total RNA was isolated with the RNeasy mini kit (QIAgen, Hilden, Germany). The cells were lysed with Qiazol at 24 and 48 hrs after exposure. The lysates from the different treatment and time points were frozen at −80 °C and later randomized for RNA extraction. RNA was quantified using a NanoDrop ND1000 (NanoDrop Technologies, Wilmington, DE, USA). The RNA quality was assessed with an Agilent 2100 Bioanalyzer (Agilent, Wokingham, UK). Samples with a RIN number greater than 8 were retained for subsequent analyses.

### RNA-seq

The Stranded Total RNA libraries were prepared in accordance with the Illumina® TruSeq Stranded Total RNA Sample Preparation guide (Illumina®, San Diego, CA, USA) with Ribo-Zero Human/Mouse/Rat for Illumina Paired-End Multiplexed Sequencing. The rRNA was selectively depleted using rRNA removal beads. The remaining RNAs were fragmented and primed for double stranded (ds) cDNA synthesis. The adapters and indexes were ligated to the end of the ds cDNAs and amplified by PCR for 15 cycles. The libraries were QCed on the Agilent BioAnalyser using a DNA 1000 chip to check the quantity and size distribution of the cDNA fragments. The samples were randomized and pooled in 8 libraries and validated using the Agilent 2100 BioAnalyzer on a High Sensitivity Chip (Agilent, Wokingham, UK). The pools were loaded at a concentration of 8 pM onto a total of 8 High Output flow cells on the Illumina® NextSeq 500 (Illumina®, San Diego, CA, USA). Samples were sequenced using 150 bp pair end runs at a target depth of 80 million reads per sample. Raw FASTQ sequence files can be found on NCBI-SRA at: https://www.ncbi.nlm.nih.gov/bioproject/PRJNA356697, SRP096285).

The FASTQC RNA-seq reads were aligned to the human genome (GRCh38) using STAR aligner^47^. The number of mapped read-pairs were counted using cufflinks based on the GENCODE v23 annotation^48^. The raw read counts were transformed using voom^49, 50^, TMM-normalized^51^ and QC-analysed using the arrayQualityMetrics package in Bioconductor^52^. Samples were scored based on 3 parameters: maplot, boxplot, heatmap. A sample was classified as an outlier if it failed two or more of the objective arrayQualityMetrics parameters.

### qPCR

cDNA synthesis was performed using the high-capacity RNA-to-cDNA Reverse Transcription Kit from Applied Biosystems (Foster City, CA, USA). qPCR was performed using Custom TaqMan array 96-well plates and TaqMan Fast Universal Master mix II with UNG and run on a fast PCR 7500 (Applied Biosystems, Foster City, CA, USA). Ct-values were normalized to GAPDH and relative quantification was analysed using the 2^−ΔΔCt^ method^53^. The genes for qPCR were selected form the list of differentially expressed genes identified in the RNA-seq data. For each treatment (3R4F 1/30, e-cigarette 1/3 and 1/7) a set of 20 genes was selected based on fold change, pFDR significance, plus supporting literature information when available. The list of genes is given in Supplementary Table S20.

### Statistics

For nicotine, pairwise comparisons were assessed using an unpaired t-test. Multiple comparisons between the treatments were performed using a Dunnett's test for TEER, CBF, and immunohistochemistry. The data was Log transformed to correct for unequal variance.

For the RNA-seq data analysis, the assessment was performed using linear modelling with the air-control exposure as reference while adjusting for relevant factors including exposure run, NGS run, treatment and time point. Subsequently, empirical Bayesian analysis was applied including p-value adjustment for multiple testing, which controls for pFDR^54^. For each comparison, the null hypothesis was that there was no difference between the groups being compared. The Bioconductor package Limma was used^50^. The primary output from the statistical analysis is a set of fully annotated (when available) lists of genes differentially expressed in the comparison of interest.

For qPCR, the Ct values were normalized against GAPDH to obtain ∆Ct. Statistical analysis, including PCA plots and Volcano plots were performed using JMP_Genomics6.0 (SAS Institute Inc, Cary, NC, USA).

### Genesest and GO functional annotation analyses

A collection of 131 genesets obtained from lists of pathway-focused arrays was used to assess the fold-changes for loci significant at pFDR < 0.05 in one or more of the comparisons and presented as heatmaps. The gene lists were obtained from the SABiociences website^55^ on July 5^th^ 2013.

Significant genes from the treatments adjusted for time and filtered at pFDR < 0.05 for e-cigarettes and a pFDR < 0.01, fold change [FC] > 2 for cigarettes were analysed for enrichment of GO terms across all three GO ontologies (biological processes, cellular components and molecular functions) using a hypergeometric test. The top 10 most significant (p-values) GO terms for each treatment have been represented in a GO circle plot^56^.

The diseases and functions tool from IPA ingenuity® (QIAgen, Redwood City, CA, USA) was used to conduct downstream causal reasoning predictions and derive associated activation z-scores. The z-scores were calculated according to Kramer *et al*.^35^ and using the gene list from the time adjusted contrasts at pFDR < 0.01 [FC] > 2 for 3R4F smoke (1/30 dilution) and pFDR < 0.05 for e-cigarette aerosol at 1/3 and 1/7 dilution. The diseases and functions tool from IPA ingenuity® (QIAgen, Redwood City, CA, USA) was used to conduct downstream causal reasoning predictions and derive associated activation z-scores. The z-scores were calculated according to Kramer *et al*.^35^ and using the gene list from the time adjusted contrasts at pFDR < 0.01 [FC] > 2 for 3R4F smoke (1:30 dilution) and pFDR < 0.05 for e-cigarette vapor at 1/3 and 1/7 dilution.

## Electronic supplementary material

Supplementary Figures S1 to S2

Supplementary Tables S1 to S9

Supplementary Tables S10

Supplementary Tables S11 to S21
