# Supplementary material for: Reduced biological effect of e-cigarette aerosol compared to cigarette smoke evaluated in vitro using normalized nicotine dose and RNA-seq-based toxicogenomics
Source: Sci Rep. 2017 Apr 18;7:888. doi: 10.1038/s41598-017-00852-y (PMC5429854; doi:10.1038/s41598-017-00852-y)
Supplement: Supplementary file 1 — Supplementary Figures S1 to S2 [file 41598_2017_852_MOESM1_ESM.pdf]

Reduced biological effect of e-cigarette aerosol compared to cigarette smoke evaluated *in vitro* using normalized nicotine dose and RNA-seq-based toxicogenomics

Linsey E Haswell[1], Andrew Baxter[1], Anisha Banerjee[1], Ivan Verrastro[1], Jessica Mushonganono[1], Jason Adamson[1], David Thorne[1], Marianna Gaça[1] & Emmanuel Minet[1]*

[1]British American Tobacco R&D Centre, Regents Park Road, Southampton, SO15 8TL UK

Supplementary Figure S1: Cilia beat frequency (CBF) immediately after aerosol exposure. Single value plots for CBF(s) measured in each insert and for each exposure run. The measurement was performed immediately after the aerosol exposure in all the available cell inserts grouped by treatment. The grand mean is shown by the dotted horizontal line. No statistical difference at $p<0.05$ was observed.

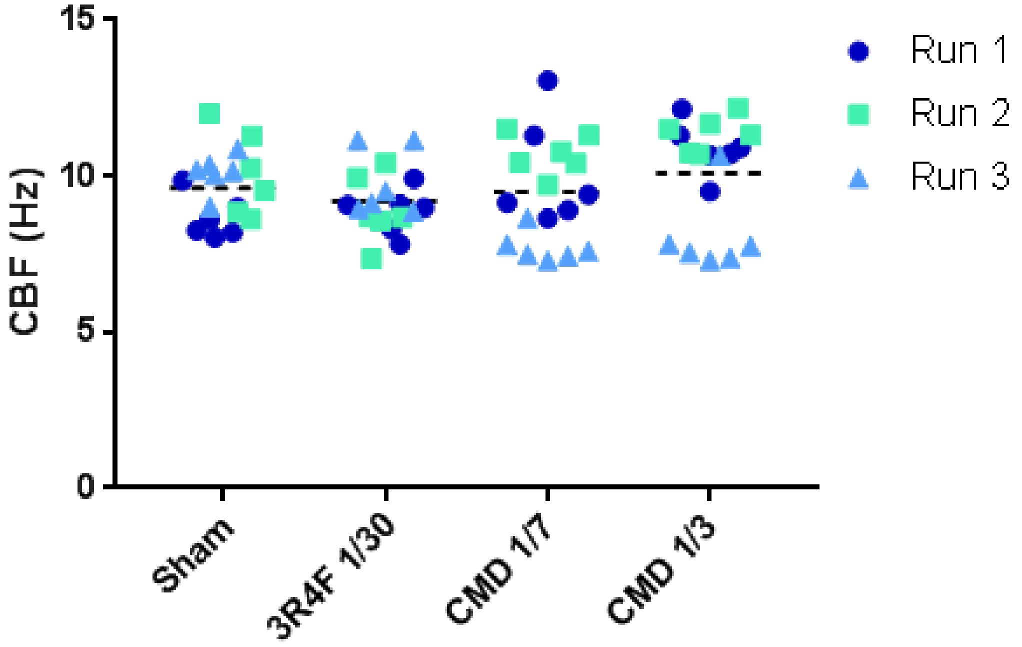

Supplementary Figure S2: Venn diagram (**A**) and table (**B**) showing the differentially expressed genes overlapping between treatments. The gene list was obtained using the pFDR and fold change criteria as shown on the figure. The dataset that were used to select those genes is after adjustment for time (24hrs+48hrs).

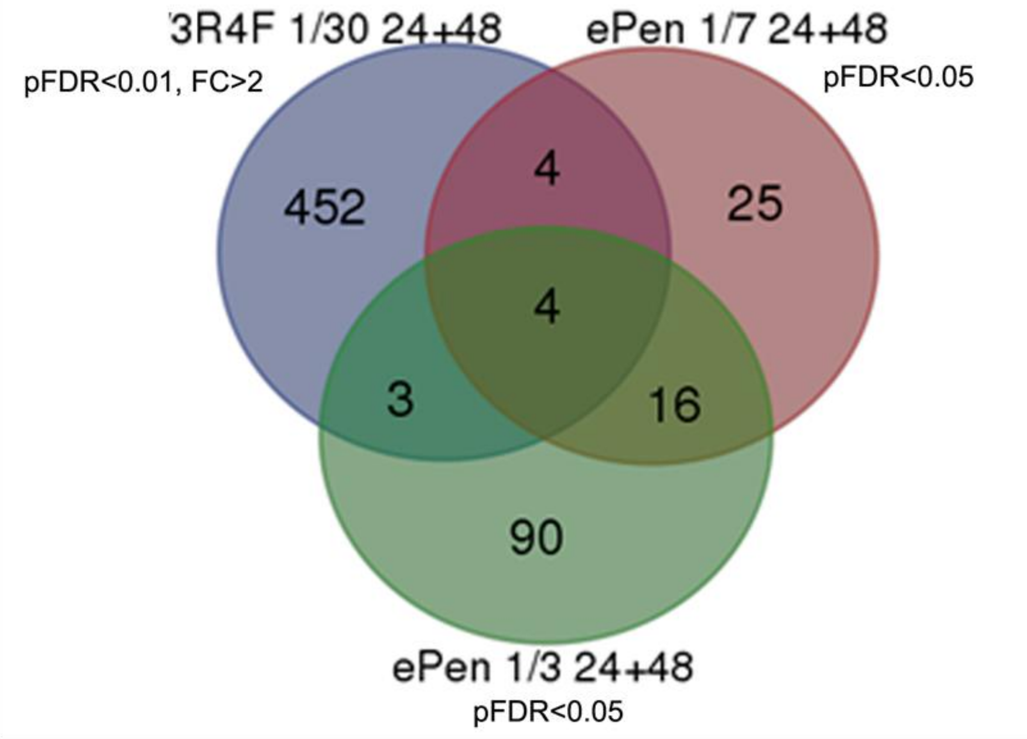

TREATMENTS	GENE OVERLAPS
3R4F 1/30, ePen 1/7, ePen 1/3	WNK2, MAN1C1, HR, SERPINB3
3R4F 1/30, ePen 1/7	RYR3, FTH1, NEU4, TFRC
3R4F 1/30, ePen 1/3	TNS3, ANPEP, 1 non coding RNA species
ePen 1/7, ePen 1/3	PTN, AZIN1, HSD17B2, CHL1, ALS2CL, MT1E, ADH6, RNF152, FAIM2, DDIT4L, HMGN4, SLC4A4, FBP1, ENPP3, FGF14, 1 non coding RNA
